# HIV/AIDS awareness and risk behaviour among pregnant women in Semey, Kazakhstan, 2007

**DOI:** 10.1186/1471-2458-8-295

**Published:** 2008-08-22

**Authors:** Emma Sandgren, Sofia Sandgren, Marat Urazalin, Rune Andersson

**Affiliations:** 1 Department of Infectious Diseases, Sahlgrenska Academy at Gothenburg University, Sweden; 2 Semipalatinsk State Medical Academy, Semey, Kazakhstan; 3 Research and Development Centre, Skaraborg Hospital, Skövde, Sweden

## Abstract

**Background:**

Central Asia has one of the most rapidly increasing HIV prevalence in the world. The aim of this study was to evaluate current knowledge, risk behaviour and attitudes to voluntary counselling and testing concerning HIV/AIDS among pregnant women in Semey, Kazakhstan.

**Methods:**

We collected 226 questionnaires in a consecutive sample from a population on 520 pregnant women. The results were related to ethnicity, age and education level.

**Results:**

Ninety-six percent had heard about HIV.

Positive findings were that 89% and 86% of the women were aware of the two main routes of transmission: sexual intercourses without a condom and sharing needles while injecting drugs. The women had first heard about HIV/AIDS through the media with, 52%, and at school with 40%. Only 46% and 68% of the women pointed out breastfeeding and mother-to-child transmission during pregnancy or delivery as routes of transmission. Eighty-three percent were prepared not to breastfeed their baby if they were found to be HIV positive. Slightly more, 86%, accepted the need to take medicine, but fewer women, 68%, were positive to Caesarean section. Negative findings were that only 28% answered that there are ways to protect oneself against sexually transmitted HIV/AIDS and specified that this was condom use.

**Conclusion:**

The pregnant women in Semey have poor knowledge about specific mother-to-child HIV transmission and do not know about the means of reducing mother-to-child HIV infection. The information in the public health program needs to be improved. However, most of the women in Semey were positive to prevention strategies for mother-to-child transmission after hearing about it.

## Background

Today about 33 million people are living with HIV. Of them 15 million are women and 2.5 million children under the age of 15 [1].

Vertical transmission of HIV from mother-to-child accounts for the vast majority of the infections among the children. Mother-to-child HIV transmission occurs intrauterine, intra-partum and during breastfeeding. Without antiretroviral treatment, the risk of an infected woman transmitting the virus to her child is between 16 and 40%. Breastfeeding contributes at least a 10% risk of transmission [2]. Timely administration of antiretroviral drugs to the HIV infected pregnant woman and her newborn significantly reduces the risk of mother-to-child transmission [3]. With antiretroviral treatment resulting in low viral load, no breastfeeding and elective Caesarean the HIV transmission rate to the child can be reduced to 0–2% [4]. Elective Caesareans are most important for women with high HIV viral loads at the time of delivery.

Pregnancy has not been proven to have any negative effects on women with asymptomatic HIV infection [5]. It seems that advanced HIV infection, however, can increase the risk for spontaneous abortion or premature birth [2].

In Kazakhstan, 16 500 people are estimated to live with HIV/AIDS which means a prevalence of 0.1–0.2% [6]. The HIV epidemic is strongly concentrated to vulnerable populations, like injecting drug users, sex workers and prisoners. In addition to these groups, the epidemic is spreading among other vulnerable groups like youth, migrants and truck drivers. About three-quarters of the new HIV diagnoses is among unemployed people. Currently the two main routes of HIV transmission in Kazakhstan are injecting drug use and sexual transmission.

Because of the increased involvement of women and children in drug use and trafficking, it's likely that the HIV prevalence in these groups will rise. There are about 20 000–50 000 female sex workers, of whom 30% are intravenous drug users [7]. According to WHO/UNAIDS report in March 2006, there were 1500 people in need of antiretroviral drugs in Kazakhstan at the end of 2005. Just 15%, of those in need, received treatment [8].

In recent years efforts has been made to cover all pregnant women with full prevention services and the mother-to-child prevention is a big part of the Kazakhstan HIV programme [6]. In average the number of births in Kazakhstan are 237 000 per year. In 2005 the antenatal care coverage were estimated to 91% and the number of women counselled on prevention of mother-to-child transmission (PMTCT) services were 129 706. The number of HIV infected women was estimated to fewer than 500 and of those 47 (9%) received antiretroviral therapy for PMTCT [9].

The aim of the study was to evaluate current knowledge, risk behaviour and attitudes to voluntary counselling and testing concerning HIV/AIDS among pregnant women in Semey, Kazakhstan. This is important, considering that the gateway for prevention of mother-to-child transmission is voluntary counselling and testing for HIV.

## Methods

The study was conducted between June 14 and July 25, 2007 in Semey, Kazakhstan. We collected 226 questionnaires from a consecutive sample of pregnant women attending four different antenatal clinics at different parts of the city. During a year there are around 1000 pregnancies in Semey followed at 23 antenatal clinics. At the included clinics 520 pregnant women were registered at the time of the study.

A questionnaire was designed to obtain three areas of interest: sociodemographic characteristics, general awareness of HIV/AIDS and attitudes and risk behaviour. The questionnaire starts out with the sociodemographic questions, like age, educational and employment level for the woman and her husband, parity, socio-economic status, religion and ethnical group. In the next part, general awareness of HIV/AIDS follows questions about if the woman has heard about HIV/AIDS before, and in case of, how she first heard about it, knowledge of route of transmission, knowledge of HIV prevention and symptoms. The third part includes questions concerning if they wanted to be tested and if they wanted more children or would be prepared not to breastfeed their baby, to take pills and deliver through Caesarean to prevent HIV transmission to their baby, given they were HIV positive. In addition, the third part includes questions about whom they would inform if they were tested positive, from whom they would get support and finally questions concerning use of condoms, sexual contacts and intravenous drug use.

The questionnaire contained three questions about condom use. The first and second questions about use of condom in steady relationship respectively with casual partners were designed to assess the women's actual practice of condom. The third question concerning responsibility for condom use is a question about the women's attitude.

The questionnaires were translated to Kazakh and Russian and the women were given questionnaires in the language they preferred.

The women were informed that their participation and the completion of the questionnaire were entirely voluntarily and that they were free not to answer the questions they found too private. The information given would be stored confidentially and no names or identifying information would appear in publications. A verbal consent was obtained for each participant. In addition to the questionnaires, we interviewed 21 of the study participants. In all interviews an interpreter was necessary; because of this a local student was always present to help us to ask questions and to take notes.

Open-ended questions were used and the women were encouraged to give as much information as they could. We were careful not to suggest answers or to ask leading questions. The questions were clustered around the areas: knowledge of differences between HIV and AIDS, living with HIV/AIDS, attitudes to people living with HIV/AIDS and in particular knowledge about mother-to-child transmission and attitudes to voluntary counselling and testing.

### Ethical considerations

The participation in the study was voluntary with informed consent. The questionnaire was anonymous with no registration of names, medical data or other personal information. In our manuscript it is not possible to identify individual patients. We have a written decision from the Head of the Ethical Committee at Semey, Kazakhstan that no further ethical review is required. With the anonymous design and no registration of personal sensitive data, the study does not need to be reviewed by the Swedish Ethical Committee according to their rules. No information was collected from the women who refused to participate.

## Results

### Study population

A total of 226 pregnant women participated in our study. Their ages ranged from 18 to 47 years, with a mean age of 26.8 and a median age of 25. Thirteen women did not answer the question about their age; therefore these women were excluded when we referred our results to age. Parity ranged from 0 to 4 children, with a mean of 0.68 and a median of 1 child. Eighteen women did not answer the question about parity.

Of the 226 women, 76.2% (170/223) were Kazakh, 18.4% (41/223) Russian, 2.2% (5/223) Tatar, 1.8% (4/223) German and 1.3% (3/223) others. The three women who did not answer the question about their ethnicity were excluded when we referred our results to ethnicity. Religion is closely linked with ethnicity. Of the Kazakh women who answered the question about religion, 100% were Muslim. Among the Russian women who answered the question about religion, 93% were Christian Orthodox. Of the whole group 79.7% (173/217) were Muslim, 18.9% (41/217) Christian Orthodox and 1.4% (3/217) other. No significant association was found between occupation and ethnic origin. When it comes to education among the women we refer to additional file 1.

Russian women significantly more often attended Special College (66%; 95% CI 51–80%) compared to 35% (95% CI 28–41%) for Kazakh women. Kazakh women had a tendency to more often attend University/Institute/Academy.

### General knowledge

Ninety-six percent (215/225) of the women had heard of HIV/AIDS.

The majority, 52.1% (111/213), of the whole group had first heard about it from the media, 40% (85/213) knew it from school, 2.8% (6/213) from their parents, 1.9% (4/213) from friends and 3.3% (7/213) from other sources. Two women did not answer this question. The older women (> 30 years old) had significantly more often, 80% (95% CI 68–91), heard about HIV/AIDS from the media than the younger women at age 20–30 on 47% (95% CI 39–55). In contrast the younger women (age < 20 and 20–30), had significantly more often heard about the disease at school, 64% (95% CI 35–92) for women age < 20 and 46% (95% CI 37–54) for women age 20–30, as compared with women with age over 30, for whom the corresponding figure was 16% (95% CI 6–27). Only one out of the 56 women who correctly answered yes to the statement that there are differences between HIV and AIDS was able to specify those differences, but the 21 women we interviewed with open questions were better at specifying the differences between HIV and AIDS. Among those who specified, many answers were unclear and difficult to interpret. However, we did obtain some more or less correct answers like: "HIV is the causative agent of the disease AIDS", "HIV means you are a carrier, while AIDS means you are sick" and "with HIV you live longer than with AIDS".

### Knowledge about transmission and symptoms

To the open question regarding the main way HIV/AIDS is spread from one person to another many women gave several answers, the most common being "sexual contact" given by 76% (122/160). The second most common answer was blood transfusion, with 30% (48/160). Nineteen percent (30/160) wrote intravenous drug use/needle sharing and 3% (5/160) wrote they didn't know.

To discover misconceptions about transmission about HIV/AIDS the women were asked to include/exclude ways that HIV/AIDS can or not can be transmitted. These answers, with reference to the women's level of education [see Additional file 2]

In general women, with higher levels of education were better than women with low education at correctly excluding and including transmission routes of HIV/AIDS. The differences were significant for "shaking hands/hugging/living in the same house", "changing clothes with someone who has HIV/AIDS", "sexual intercourses with condom", and "sharing needles while injecting drugs". For details, see Additional file 2.

As many as 76% (157/206) answered correctly, with no, to the statement you can not tell, by looking at a person, whether he/she is infected with HIV/AIDS. Younger women (age < 20) significantly more often, 100%, answered no to the statement that by looking at a person you can see if he/she is infected with HIV/AIDS, as compared with women aged 20–30 years, 76% (95% CI 69–83) and women aged over 30 years, 70% (95% CI 57–84).

The open question "Do you know any symptoms of HIV/AIDS" was left blank by 111 women. Of the responders, 66% (69/104) answered no. Among the women who could mention any symptoms the most common answers were fever/sub febrile 37% (13/35), loss of immunity 34% (12/35), weakness 29% (10/35) and cahexia/weight loss 17% (6/35). When the women's ability to mention symptoms of HIV/AIDS were refered to educational level, the women with low education levels significantly more often, 95% (95% CI 85–100), answered that did not know any symptoms, as compared with women with higher levels of education, 60% (95% CI 50–70).

### Knowledge about treatment

A total of 14% (28/201) believed that there is a medicine to cure HIV/AIDS, and nearly the same number, 15% (30/202) stated that there are other ways to cure HIV/AIDS. Many more, almost one out of three, stated that there are possible ways to slow the progression of the disease.

### Having HIV/AIDS

Of the whole group 41% (88/215) of the women wanted to be tested for HIV/AIDS. Fifty-nine percent (124/211) of the women said they would tell the healthcare personnel if they were found to be HIV positive, 35% (74/211) that they would tell their mothers and 32% (68/211) their partners. Fifteen women left the question blank. Statistically significantly more Russian women, 51% (95% CI 36–66) said they would tell their partner as compared with the Kazakh women, 26% (95% CI 19–33), but on the other hand there was a tendency among the Kazakh women to say more often that they would tell their friends than among the Russian women. There was also a tendency for the Russian women to say more often that they would tell their mothers, but the difference was within the margins of error.

Regarding from whom the women thought they would get support if they were found to be HIV positive, the far most common answer was mother, with 51%. Only 45% thought that they would get support from healthcare personnel, and even fewer, 21%, said they would get support from their fathers, partners 33% or friends 23%. Sixteen women (7%) did not answer the question. A comparison between Kazakh and Russian women showed that more Russian women expected to get support from mothers and partners, while Kazakh women more often mentioned friends and healthcare personnel. Note that the women could tick several alternatives.

### Attitudes to PMTCT, prevention of mother-to-child transmission

Seven women said they would want to have more children even if they were found to be HIV positive. Among the women who said no to have more children 82% (176/216), the main reason given was the risk of infecting the baby. Significantly more highly educated women said no, 87% (95% CI 81–92), to having more children as compared with the less educated women, 66% (95% CI 53–79). Of the responders 83%, were prepared not to breastfeed their baby if they were found to be HIV positive. Somewhat more, 86%, were prepared to take medicine, but fewer women, 68%, were prepared to accept Caesarean section to prevent mother-to-child transmission.

### Risk behaviour and protection

Ninety-six percent (179/186) had had one sexual partner during the last sixth months. The three questions about condom use were as follow: "Use of condom in steady relationship?", "Use of condom with casual partners?" and "Who has the responsibility for condom use?" Of the women who answered the first question 21% (42/201) answered always, 40% (80/201) never and 39% (79/201) sometimes. Of the responders to the second question, as few as 57% (78/137) answered always, 34% (47/137) never and 9% (12/137) answered sometimes.

About two thirds, 68% (141/206), of the women stated that men and women have equal responsibility to make sure a condom is used during sexual intercourses. Of our responders 65% (134/206) answered that there are ways to protect oneself against sexually transmitted HIV/AIDS. Of the women who answered yes to this question, 55% (74/134) specified ways to protect oneself. Note that the women were allowed to give more than one way of protection.

The far most common answer was to use condoms, 82% (61/74). Other answers were "avoiding casual sexual contacts" and "use of clean syringes". When the women were asked if they had sufficient information to protect themselves against HIV/AIDS, 42% (89/211) answered no and nearly the same number, 42% (88/211) answered yes. Russian women answered to a significantly higher degree 63% (95% CI 49–78) that they had sufficient information compared to Kazakh women 36% (95% CI 28–43).

### HIV epidemic

Of our responders 47% (93/198) answered that certain groups of people are more often infected with HIV than others. The far most common group was drug addicts/intravenous drug users, mentioned by 88% (49/56) of the responders. Other groups, given by less than 10% each, were people with many sexual partners, prostitutes, homeless people, homosexual people, medical people, people with low social status, and people without knowledge about the disease and blood donors/recipients. One misconception was that 11% (6/56) of the responders answered that people with poor immune defence systems/organisms are more often infected.

## Discussion

The HIV epidemic in Central Asia is continuing to spread and has reached Kazakhstan. The number of reported HIV cases is still relatively low and mainly concentrated to vulnerable populations such as intravenous drug users and sexual workers. To prevent future spread of the HIV epidemic and to prevent stigmatization in Kazakhstan and other countries in Central Asia it is important to evaluate HIV knowledge and to educate the population with correct information.

### Knowledge

It is reassuring to see that only ten women out of 226 did not know there was a disease called HIV or AIDS. This corresponds to the findings in several other studies concerning awareness about HIV/AIDS among pregnant women from India [10], China [11], Papua New Guinea [12] and Ghana [13]. Compared with the women in Aksu, northwest China, the women in Semey had more often heard of about HIV/AIDS. 95% (95% CI 92–98) compared to 85% (95% CI 80–89) in Aksu. It is positive that the media are a major source of information, exactly as in the Aksu study and the Papua New Guinea study [12]. That a majority of the women are aware of a disease called HIV/AIDS and that media are a major source of information shows that the HIV epidemic is discussed in public and that the media are an efficient way of spreading information. In addition illiteracy is rare in Kazakhstan, which facilitate for the women to take share of the given information.

However, it is also important that the information is correct, otherwise misconceptions may lead to further stigmatization. Because the women had difficulties distinguish HIV from AIDS and only 16% could mention symptoms of HIV/AIDS, we conclude that the women's knowledge in general was superficial with little understanding of the details and the nature of the disease. We were pleased to find that as many as 76% answered that you can not tell by looking at a person if she/he is infected with HIV/AIDS. The younger women, as compared with the older women, significantly more often answered no to this question. This may be explained by the fact that nowadays students got a lot more information at school.

The women in Semey were, as compared with the women in Aksu, significantly better at pointing out the high risk behaviour "sexual intercourse without condom", 89% (95% CI 85–93), as compared with 73% (95% CI 67–78) in Aksu and "sharing needles while injecting drugs", 86% (95% CI 81–90) compared to 55% (95% CI 49–61) in Aksu as routes of transmission. This is positive because these two routes are the most important routes for the general population to be aware of.

The finding that pregnant women in general are aware of the two main routes: sexual intercourse without a condom and sharing needles while injecting drugs, of HIV transmission is in agreement with previous studies [11-13], and might not be so surprising since media focus on these two main routes in their information to the public. There were more difficulties in excluding incorrect routes of transmission. Almost one fifth though that kissing could be a route of HIV transmission and only 44% answered correctly that HIV can not be spread by mosquitoes. This is slightly lower than in the Hong Kong study where 57% answered to mosquitoes as vectors of HIV [14]. The misconception about mosquitoes as a transmission route has also been seen in other similar studies [10-12] and might not be very surprising, since HIV is a blood-borne disease. The pregnant women in our study, like the women in a similar study conducted in the province Yunnan, China [11] excluded, to a higher, but not satisfactory extent daily domestic contacts such as eating from the same plates and cups, shaking hands, hugging, living in the same house and changing clothes with someone who has HIV/AIDS as possible routes of transmission. The fact that a high proportion of the women responded "don't know" in addition to the women with incorrect answers further illustrates their limited knowledge.

Our conclusion is, as in the study conducted in Aksu, that most of the women know that HIV is a sexually transmitted disease, but there are still people who have poor knowledge of HIV/AIDS and people with misconceptions about how it is spread. These misconceptions may influence the dissemination patterns of HIV and increase the stigmatization of the HIV positive, as well as giving rise to misguided fears. It is essential to continue to raise the level of knowledge of HIV/AIDS through campaigns in the media and at schools. One thing that needs to be communicated is that there is no medicine to cure a HIV infection, only 40% (95% CI 33–47) of the women answered correctly that this was the case. This may reflect that the information about HIV/AIDS in the media focus on risk behaviour and not on possible treatment. In addition the prevalence of HIV is low in Kazakhstan and few of the women participating in our study had met or knew anyone with the infection i.e. they have never come across persons under treatment for HIV/AIDS. This is significantly lower than the corresponding figure for the women in the Hong Kong study, where 79% (95% CI 73–84) answered no to the statement that there are medicines available to cure HIV/AIDS. The pregnant women in Ghana also significantly more often, 90% (95% CI 86–93), answered no to the statement that there are medicines/treatment available to cure HIV/AIDS. That significantly more women in Ghana know that there are no available treatment to cure HIV/AIDS can be explained by that the prevalence is higher (3.1% in 2003, according to CIA homepage) in Ghana and therefore more women have come across friends or relatives under treatment.

### Having HIV/AIDS

There were fewer women in our study, who would take a HIV test if it was provided, 41% (CI 95% 34–47) as compared with 77% (95% CI 71–83) in the Hong Kong study. Greater willingness among pregnant women to take an HIV test is also reported from several other studies [10-13,15,16]. The low prevalence of pregnant women willing to take an HIV test in Kazakhstan is not positive and can be explained of that Kazakhstan has a high coverage, 91% in 2005 of antenatal care [9]. Therefore, many of the women participating in our study already had been tested for HIV/AIDS in the beginning of their current pregnancy. Considering this the high rate of women answering no to have an HIV test may not indicate unwillingness, instead it may indicate the fact that they already have been tested. When it comes to informing those close to themselves a majority of the women said they would inform the healthcare personnel if they were found to be HIV positive. This indicates that many of the women have put their confidence in the health care system, which increases the system's chance to stop further spread of HIV and to give the infected women treatment. Only one out of three women felt confident enough to inform and get support from their husband, which is a problem, since informing one's partner is an important issue in the prevention of further spread. The study conducted in Aksu showed similar results.

### Knowledge and attitudes toward PMTCT, prevention of mother-to-child transmission

The women in Semey had limited knowledge about mother-to-child HIV transmission, considering that prevention of mother-to-child transmission of HIV is a large part of the Kazakhstan HIV program. Both the questionnaires and the interviews confirmed this lack of awareness. Although 68% (95% CI 62–75) knew that HIV could be transmitted to the foetus by an infected mother during pregnancy and delivery, only 46% knew that breastfeeding can be a route of transmission. An explanation can be that transmission from mother to child not is a main route in Kazakhstan and therefore the women have not been reached on prevention strategies and information about this transmission way. A comparison with the women in the Hong Kong study shows that the Hong Kong women were significantly better at pointing out pregnancy 97% (95% CI 94–99) and delivery 91% (95% CI 86–94) as possible routes of mother-to-child transmission, but when it came to breastfeeding around the same percentage of women in both our study and the Hong Kong study identified this as a route of HIV transmission. The uncertainty about mother-to-child transmission as a route of HIV transmission has also been identified in other studies [11,13,15,16]. In contrast to the studies mentioned above, two similar studies conducted in India [10] and Papua New Guinea [12] reports a higher percentage, 80% and 69% respectively, of women who knew about breastfeeding as a route of transmission of HIV from mother to child. When it comes to knowledge about pregnancy and delivery as routes of transmission, around the same proportion of pregnant women in India and Papua New Guinea, as in the Hong Kong study [14], knew about these routes.

Even if the women in Semey seem to have poor knowledge about routes of mother-to-child HIV transmission, it is positive to see that only seven out of 226 women said they would want to give birth to more children if they were found to be HIV positive. However, in comparison to the women in the Aksu study this result is not satisfactory. The women in the Aksu study stated significantly more often, 97% (95% CI 95–99), that they would not have more children, as compared with 82% (95% CI 77–87) of the Semey women. This may be a result of that a family is central for the women in Kazakhstan and the women may have difficulties to accept not to have any more children, in contrast the women in China are not allowed to give birth to more than one child according to their law. It is also positive that the vast majority of the women were prepared not to breastfeed (the same was seen in the Aksu study) and to take medication to prevent mother-to-child transmission. It is therefore of great importance that HIV positive mothers get help both financially and with information so that they actually can take the necessary medication and not breastfeed.

The majority of the women were prepared to accept Caesarean section if being HIV positive and being recommended this operation to reduced mother to child transmission. One out of four women answered "don't know" to this question. A possible explanation for why just 68% of the women answered they were prepared to have Caesarean section could be that the women don't know what the term "Caesarean section" means. Another explanation may be that some women consider it too expensive.

### HIV epidemic

The women in Semey are not a group with generally high risk behaviour. Many women are aware that intravenous drug use is a problem in Kazakhstan, but few of our responders had been in contact with drug use personally. Slightly less than half our responders wrote that certain groups are more often infected with HIV/AIDS than others. Of those who specified their answers, 11% answered that people with poor immune defence systems/organisms were more often infected, a misconception probably attributable to confusion with the information that HIV causes poor immune defence system. The high level of condom use in steady relations may be reflecting that the majority of the participating women were well educated and living in a city. An average family in the cities has lower number of children than an average family in rural villages.

## Conclusion

Almost all the women in our study had heard of a disease called HIV/AIDS. The women's knowledge, however, was somewhat superficial and many could not specify their answers. Still, most of the women managed to identify sexual contacts and intravenous drug/needle sharing as main routs of transmission. The knowledge among pregnant women in Semey was similar to the knowledge among pregnant women in Aksu, northwest China.

The media were a main source of information, showing that the HIV epidemic is discussed in public and that the media are an efficient way of disseminating information. However, it is important that the information is correct, including that social contacts are not a risk, otherwise misconceptions may lead to further stigmatization.

One conclusion is that pregnant women in Semey have poorer knowledge than the pregnant women in Hong Kong about specific mother-to-child HIV transmission and do not know about the means of reducing mother-to-child HIV infection. However, most of the women in Semey were positive to prevention strategies for mother-to-child transmission. It is therefore important that testing and counselling for both men and women are available free of charge. Individuals who test positive need to be supported to prevent further risk behaviour, and HIV positive pregnant women should be offered antiretroviral treatment and help to shorten the breastfeeding period, to prevent mother-to-child transmission.

It is noteworthy that only a minority of the women specified condom use as a means of protection. There is room for improvement in this respect, and the importance of the use of condoms cannot be underestimated, since condom use is one of the most important means of preventing extension of the HIV epidemic.

## Competing interests

The authors declare that they have no competing interests.

## Authors' contributions

All the authors have together participated in the design of the study. ES and SS have made the interview, distributed the questionnaires and registered the data with the supervision by MU. ES, SS have made the analysis of the data together with RA and the statistician Salmir Nasić. RA, MU, ES, SS have participated in interpretation of data and preparation of the manuscript. All the authors have read and approved the final manuscript.

## Pre-publication history

The pre-publication history for this paper can be accessed here:



## Supplementary Material

Additional file 1 Education among the pregnant women in Semey related to ethnic group. The data provided present the level of education among the ethnic groups with 95% confidence intervals. Click here for file

Additional file 2 Knowledge of HIV transmission routes according to educational level and total. The data provided present the level of HIV knowledge related to level of education and ethnic group. The results are presented in a table with 95% confidence intervals. Click here for file
