# Supplementary material for: HIV/AIDS awareness and risk behaviour among pregnant women in Semey, Kazakhstan, 2007
Source: BMC Public Health. 2008 Aug 22;8:295. doi: 10.1186/1471-2458-8-295 (PMC2543023; doi:10.1186/1471-2458-8-295)
Supplement: Additional file 1 — Education among the pregnant women in Semey related to ethnic group. The data provided present the level of education among the ethnic groups with 95% confidence intervals. [file 1471-2458-8-295-S1.pdf]

	Total, N=226		Ethnical group, N= 223, 3 didn´t answer				
			Kazakh, N=170		Russian, N=41		Other, N=12
**Women´s education (N=226)**	%	CI 95%	%	CI 95%	%	CI 95%	%
Never been to school	0.9	0.2-3.2	1.2	0.3-4.2	0	-	0
Not finished nine-year compulsory school	4.0	1.5-6.6	3.0	1.3-6.7	7.3	2.5-19.4	8
Nine-year compulsory school	19.3	14.1-24.5	21.3	15.1-27.5	7.3	2.5-19.4	17
Special college	40.8	34.4-47.3	**34.9**	**27.7-42.1**	**66.0**	51.3-80.4	42
University/Institute/Academy	35.0	28.7-41.2	39.6	32.3-47.0	20	7.4-31.6	25
Other	0		0		0		8

Additional file 1.

Education among the pregnant women in Semey related to ethnic group.
