# Supplementary material for: HIV/AIDS awareness and risk behaviour among pregnant women in Semey, Kazakhstan, 2007
Source: BMC Public Health. 2008 Aug 22;8:295. doi: 10.1186/1471-2458-8-295 (PMC2543023; doi:10.1186/1471-2458-8-295)
Supplement: Additional file 2 — Knowledge of HIV transmission routes according to educational level and total. The data provided present the level of HIV knowledge related to level of education and ethnic group. The results are presented in a table with 95% confidence intervals. [file 1471-2458-8-295-S2.pdf]

	Educational level														
	Low*, N=50					High**, N=165					All women, N=215				
**Belifs on ways of Transmission:**	Yes		No		DK	Yes		No		DK	Yes		No		DK
	%	95% CI	%	95% CI	%	%	95% CI	%	95% CI	%	%	95% CI	%	95% CI	%
Eating or drinking from the same plates and cups?	24.0	12.2-35.8	44.0	30.2-57.8	32	13.3	8.1-18.5	61.2	53.8-68.6	25.5	15.8	10.9-20.7	57.2	50.6-63.8	27.0
Shaking hands/ hugging/ living in the same house?	12.0	3.0-21.0	**46.0**	**32.2-59.8**	42	4.8	1.6-8.1	**73.3**	**66.6-80.1**	21.8	6.5	3.2-9.8	67.0	60.7-73.3	26.5
Changing clothes with someone who has HIV/AIDS?	8.0	3.2-18.8	**44.0**	**30.2-57.8**	48	4.8	1.6-8.1	**69.1**	**62.0-76.1**	26.0	5.6	2.5-8.6	63,3	56.8-69.7	26.5
Kissing?	22.0	10.5-33.5	32.0	19.1-44.9	48	18.8	12.8-24.7	48.5	40.9-56.1	32.7	19.5	14.2-24.8	44.7	38.0-51.3	38.8
Sexual intercourse without condom?	78.0	66.5-89.5	4.0	1.1-13.5	66	92.7	88.8-96.7	1.2	0.3-4.3	6.1	**89.3**	**85.2-93.4**	1.9	0.7-4.7	8.8
Sexual intercourse with condom?	10.0	4.3-21.4	**38.0**	**24.5-51.5**	18	7.9	3.8-12.0	**59.4**	**51.9-66.9**	32.7	8.4	4.7-12.1	54.4	47.8-61.1	37.2

Sharing needles while injecting drugs?	**68.0**	**55.1-80.9**	4.0	1.1-13.5	52	**90.9**	**86.5-95.3**	0.6	0.1-3.4	8.5	**85.6**	**80.9-90.3**	1.4	0.5-4.0	13.0
Breastfeeding?	36.0	22.7-49.3	12.0	3.0-21.0	28	49.1	41.5-56.7	13.9	8.7-19.2	37.0	46.0	39.4-52.7	13.5	8.9-18.1	40.5
From mother-to-child during pregnancy or delivery?	54.0	40.2-67.8	2.0	0.4-10.5	52	72.7	65.9-79.5	3.6	0.8-6.5	23.6	**68.4**	**62.2-74.6**	3.3	0.9-5.6	23.7
By mosquitos?	10	4.3-21.4	36	22.7-49.3	54	15.2	9.7-20.6	46.7	39.1-54.3	38.2	14.0	9.3-18.6	44.2	37.5-50.8	41.9
By air-drop and air-dust?	8.0	3.2-18.8	38.0	24.5-51.5	54	7.3	3.3-11.2	61.2	53.8-68.6	31.5	7.4	3.9-11.0	55.8	49.2-62.5	36.7

Additional file 2

Knowledge of HIV transmission routes according to educational level and total. Note that women answering no or no answer to if they have heard of HIV/AIDS were excluded. * Never been to school/not finished or finished nine-year compulsory school, **Special college/University/Institute/Academy, DK= don’t know or no answer.
